# Impact of Lung Disease on COVID-19 Health Outcomes in People Living With HIV

**DOI:** 10.7759/cureus.42368

**Published:** 2023-07-24

**Authors:** Taiwo O Aremu, Oluwafemi Ajibola, Joseph Akambase, Oluwatosin E Oluwole, Han Lu, Grace Hernandez, Nicholas Hable, Jennifer McKay, Modupeoluwa Owolabi, Olawale Ajibola, Kehinde O Adeyinka

**Affiliations:** 1 Environmental Health Sciences, School of Public Health, University of Minnesota, Minneapolis, USA; 2 Pediatric Hematology and Oncology, University of Minnesota Masonic Children's Hospital, Minneapolis, USA; 3 Pharmaceutical Care & Health Systems, College of Pharmacy, University of Minnesota, Minneapolis, USA; 4 Pulmonary and Critical Care Medicine, Louisiana State University Health Sciences Center, New Orleans, USA; 5 Internal Medicine, Hennepin County Medical Center, Minneapolis, USA; 6 Epidemiology and Community Health, School of Public Health, University of Minnesota, Minneapolis, USA; 7 Biostatistics, School of Public Health, University of Minnesota, Minneapolis, USA; 8 Medicine, University of Minnesota, Minneapolis, USA; 9 Internal Medicine, Saint Michael's Medical Center, Newark, USA; 10 Medicine, American University of St. Vincent School of Medicine, Kingstown, VCT; 11 Radiation Oncology, University College Hospital (UCH), Ibadan, NGA

**Keywords:** covid-19-specific mortality, all-cause mortality, people living with hiv (plwh), interstitial lung disease, pulmonary hypertension, asthma, chronic obstructive pulmonary disease (copd), pulmonary disease, coronavirus 2019 (covid-19), human immunodeficiency virus (hiv) infection

## Abstract

Introduction

COVID-19 most commonly causes pulmonary/lung infection, and these pulmonary diseases can complicate HIV infection. Underlying pulmonary diseases in people living with HIV (PLWH) could affect health outcomes if infected with COVID-19. Therefore, this study was designed to determine the impact of pulmonary diseases on the health outcomes of PLWH that were infected with COVID-19.

Materials and methods

We conducted a retrospective study to assess the impact of superimposed COVID-19 infection on pre-existing lung pathologies in patients living with human immunodeficiency virus (HIV) infection using data from the Minnesota Fairview network from January 1, 2020 to December 31, 2022. Ordinal logistic regressions were used to determine the effect of lung comorbidities on COVID-19 severity, COVID-19-specific mortality, and all-cause mortality, adjusting for patient age and gender.

Results

Two hundred sixteen PLWH tested positive for COVID-19. 24.54% of these patients had one or more pulmonary diseases, including asthma, chronic obstructive pulmonary disease (COPD), and other lung diseases (interstitial lung diseases and pulmonary hypertension). The severity of COVID-19 outcomes was evaluated by the ranking of patients’ medical records of testing positive, admitted to the hospital, being admitted to the ICU, and death. COVID-19-specific and all-cause mortality were evaluated separately. PLWH with underlying asthma or COPD was not associated with increased all-cause or COVID-19-specific mortality. Interstitial lung disease or pulmonary hypertension was significantly associated with poor health outcomes for COVID-19-specific mortality and all-cause mortality (Fisher’s Exact p-value <0.001), with ICU admissions accounting for the most impact. Using the multivariate models, interstitial lung disease and pulmonary hypertension was significantly associated with an increased risk of more severe COVID-19 outcomes and COVID-19-specific mortality (OR=6.6153, CI=2.5944, 17.0795, p-value < 0.001). Interstitial lung disease and pulmonary hypertension were also significantly associated with an increased risk of more severe COVID-19 outcomes and all-cause mortality (OR=​​5.0885, CI=2.0590, 12.5542, p-value < 0.001).

Conclusions

To mitigate the poor outcomes associated with interstitial lung diseases and pulmonary hypertension in PLWH due to COVID-19, healthcare providers must educate their patients about safety measures against the COVID-19 vaccine. They can also encourage the COVID-19 vaccine uptake among their eligible patients.

## Introduction

Lung disease, otherwise known as pulmonary disease, is a condition of public health importance, including significant morbidity and mortality rates in the United States. The pulmonary disease remains one of the leading causes of death after heart disease, cancer, coronavirus 2019 (COVID-19), accidents, and cerebrovascular diseases, accounting for about 43 deaths per 100,000 United States population [1,2].

The human immunodeficiency virus (HIV) infection, an RNA virus that destroys the T cells of the immune system, can be complicated by different types of pulmonary disease. The noninfectious pulmonary complications that have been associated with HIV include emphysema, bronchiectasis, interstitial lung diseases (ILD), pulmonary hypertension (PH), and pulmonary malignancies [3,4].

COVID-19, a disease caused by severe acute respiratory syndrome coronavirus 2 (SARS-CoV-2), most commonly causes pulmonary infection, and the underlying pulmonary disease has been associated with increased mortality in patients infected with COVID-19 infection. The risk factors associated with increased mortality in COVID-19-infected patients include dementia, age 65 or older, obesity, male sex, high oxygen requirement, chronic obstructive pulmonary disease (COPD), hypercholesterolemia, type 2 diabetes mellitus, and cardiovascular diseases [5-7].

Recent studies on people living with HIV (PLWH) suggested an increased risk for severe COVID-19 disease progression [4]. However, it is unclear if this high-risk tendency is due to the increased prevalence of comorbidities in PLWH [4]. Therefore, this study aims to explore the impact of pulmonary diseases on the health outcomes of PLWH that were infected with COVID -19.

## Materials and methods

Study design

This was a retrospective chart review designed to further characterize the impact of superimposed COVID-19 infection on pre-existing lung pathologies in persons living with HIV infection using data from the Minnesota Fairview network, Minnesota. Data were abstracted from EHR using a templated chart. Clinical data including various diagnoses of lung pathologies among others as well as demographic information were collected. We included all patients living with HIV infection who tested positive for COVID-19 (via nasal swab) and received care within the Minnesota Fairview network from January 1, 2020, to December 31, 2022. Those with incomplete data were excluded from our analysis.

Variables

All exposure variables were defined as per the ICD-10 diagnosis code and captured in the medical records of patients who had contact with the Fairview network in Minnesota. The exposure variable was HIV. This was retrieved from the repository of all HIV-positive patients that visited the hospital for whatever reason within the time period considered. The outcome variable was COVID-19, including deaths due to COVID-19 (COVID-19-specific mortality). Age and gender were considered confounding variables. Confirmed COVID-19 test results were retrieved from the electronic medical record by a Best Practices Integrated Informatics Core (BPIC) Analyst.

Statistical analysis

Descriptive analyses were conducted to describe patients’ characteristics, including age and gender, across the four groups of patients. Continuous variables (age) were presented with median and interquartile range (IQR) and were tested with the Kruskal-Wallis rank sum test. Categorical variables (gender) were presented with count and percentage and were tested with the Fisher’s Exact test. These non-parametric tests, the Kruskal-Wallis rank sum test, and Fisher’s Exact test were used in considering the imbalanced sample size among the groups.

In regression analysis, the primary outcome of interest, the severity of COVID-19-related health outcome, is characterized from patients’ medical record, ranking from the least severe with no record other than testing positive for COVID-19, followed by admitted to hospital for COVID-19, then admitted to ICU for COVID-19, and the most severe with death. We considered COVID-19-specific mortality and all-cause mortality separately in analyses. Ordinal logistic regressions were performed to analyze the effect of lung comorbidities on COVID-19 severity and COVID-19-specific mortality, adjusting for patient age and gender. Ordinal logistic regressions were also performed to analyze the effect of lung comorbidities on COVID-19 severity and all-cause mortality, adjusting for patient age and gender. Lung comorbidities/pulmonary conditions assessed include asthma, COPD, and other lung diseases (including ILD and PH) as stated in the patients' medical records. All statistical tests were two-tailed, with the significance level (alpha) set to 0.05. All data analyses were performed with the use of R software, version 4.2.1.

Ethical consideration

This study was conducted with the Declaration of Helsinki and approved by the Institutional Review Board of the University of Minnesota as a human research exempt study (IRB ID: STUDY00013254).

## Results

Among 1,279 HIV-positive patients who had visited the Minnesota Fairview Network from January 1, 2020 to December 31, 2022, 16.88% (n=216) tested positive for COVID-19, hence, were included in the analysis. The patient’s median age was 54 with IQR (42.00, 61.00) years, and the majority were males (67.59%, n=146). 24.54% (n=53) of the patients had one or more pulmonary conditions on record, with asthma accounting for the majority (n=26), followed by COPD (n=22) and then other lung diseases (n=18) including interstitial lung disease and PH.

Descriptive statistics based on COVID-19-specific mortality

The baseline patient characteristics and lung comorbidities were analyzed between the four groups of patients based on COVID-19 severity and COVID-19 specific mortality: no COVID-19-related record other than testing positive, admitted to the hospital due to COVID-19, admitted to ICU due to COVID-19, and death possibly related to COVID-19. Death possibly related to COVID-19 was identified by the doctor’s note of primary and secondary cause-of-death. Age (Kruskal-Wallis Rank Sum p-value = 0.679) and gender (Fisher’s Exact p-value = 0.326) were not significant across the four groups, suggesting that baseline patient characteristics were balanced. Among individuals with lung-related comorbidities, having one or more pulmonary conditions on record (Fisher’s Exact p-value = 0.255), COPD (Fisher’s Exact p-value = 0.597), and asthma (Fisher’s Exact p-value = 0.865) were not significant across the four groups. Having interstitial lung disease or PH was significantly different across the four groups (Fisher’s Exact p-value < 0.001), with ICU admissions accounting for the highest proportion (33.33%) (Table 1).

**Table 1:** Baseline patient characteristics for COVID-19-specific mortality COPD: chronic obstructive lung disease; ILD: interstitial lung disease; PH: pulmonary hypertension

Parameters	Overall, N = 216	No More Record, N = 161	Admitted to Hospital, N = 42	Admitted to ICU, N = 9	COVID-19-Specific Mortality, N = 4	P-value
Age	54 (42.00, 61.00)	54 (44.00, 61.00)	54 (40.50, 64.75)	60 (48.00, 63.00)	49 (48.50, 49.00)	0.679
Sex						0.326
Female	70/216 (32.41%)	54/161 (33.54%)	15/42 (35.71%)	1/9 (11.11%)	0/4 (0.00%)	
Male	146/216 (67.59%)	107/161 (66.46%)	27/42 (64.29%)	8/9 (88.89%)	4/4 (100.00%)	
Pulmonary Conditions						0.255
Yes	53/216 (24.54%)	35/161 (21.74%)	13/42 (30.95%)	4/9 (44.44%)	1/4 (25.00%)	
No	163/216 (75.46%)	126/161 (78.26%)	29/42 (69.05%)	5/9 (55.56%)	3/4 (75.00%)	
COPD						0.597
Yes	23/216 (10.65%)	16/161 (9.94%)	5/42 (11.90%)	2/9 (22.22%)	0/4 (0.00%)	
No	193/216 (89.35%)	145/161 (90.06%)	37/42 (88.10%)	7/9 (77.78%)	4/4 (100.00%)	
Asthma						0.865
Yes	26/216 (12.04%)	21/161 (13.04%)	5/42 (11.90%)	0/9 (0.00%)	0/4 (0.00%)	
No	190/216 (87.96%)	140/161 (86.96%)	37/42 (88.10%)	9/9 (100.00%)	4/4 (100.00%)	
Other lung diseases, including ILD and PH						<0.001
Yes	18/216 (8.33%)	6/161 (3.73%)	8/42 (19.05%)	3/9 (33.33%)	1/4 (25.00%)	
No	198/216 (91.67%)	155/161 (96.27%)	34/42 (80.95%)	6/9 (66.67%)	3/4 (75.00%)	

Descriptive statistics based on all-cause mortality

After replacing death possibly related to COVID-19 with all-cause mortality, age (Kruskal-Wallis Rank Sum p-value = 0.684) and sex (Fisher’s Exact p-value = 0.595) were not significant across the four groups, suggesting that baseline patient characteristics were balanced. The respective lung-related comorbidities, including one or more pulmonary conditions on record (Fisher’s Exact p-value = 0.497), COPD (Fisher’s Exact p-value = 0.862), and asthma (Fisher’s Exact p-value = 0.681) showed no significant difference across the four groups. However, having interstitial lung disease or PH was significantly different across the four groups (Fisher’s Exact p-value < 0.001), with patients admitted to ICU due to COVID-19 accounting for the highest proportion (28.57%) (Table 2).

**Table 2:** Baseline patient characteristics for all-cause mortality COPD: chronic obstructive lung disease; ILD: interstitial lung disease; PH: pulmonary hypertension

Parameters	Overall, N = 216	No More Record, N = 158	Admitted to Hospital, N = 40	Admitted to ICU, N = 7	All-Cause Mortality, N = 11	P-value
Age	54 (42.00, 61.00)	54 (43.25, 61.00)	54 (41.50, 64.25)	60 (50.50, 64.00)	49 (48.00, 66.50)	0.684
Sex						0.595
Female	70/216 (32.41%)	53/158 (33.54%)	14/40 (35.00%)	1/7 (14.29%)	2/11 (18.18%)	
Male	146/216 (67.59%)	105/158 (66.46%)	26/40 (65.00%)	6/7 (85.71%)	9/11 (81.82%)	
Pulmonary Conditions						0.497
Yes	53/216 (24.54%)	35/158 (22.15%)	13/40 (32.50%)	2/7 (28.57%)	3/11 (27.27%)	
No	163/216 (75.46%)	123/158 (77.85%)	27/40 (67.50%)	5/7 (71.43%)	8/11 (72.73%)	
COPD						0.862
Yes	23/216 (10.65%)	16/158 (10.13%)	5/40 (12.50%)	1/7 (14.29%)	1/11 (9.09%)	
No	193/216 (89.35%)	142/158 (89.87%)	35/40 (87.50%)	6/7 (85.71%)	10/11 (90.91%)	
Asthma						0.681
Yes	26/216 (12.04%)	21/158 (13.29%)	5/40 (12.50%)	0/7 (0.00%)	0/11 (0.00%)	
No	190/216 (87.96%)	137/158 (86.71%)	35/40 (87.50%)	7/7 (100.00%)	11/11 (100.00%)	
Other lung diseases, including ILD and PH						0.001
Yes	18/216 (8.33%)	6/158 (3.80%)	8/40 (20.00%)	2/7 (28.57%)	2/11 (18.18%)	
No	198/216 (91.67%)	152/158 (96.20%)	32/40 (80.00%)	5/7 (71.43%)	9/11 (81.82%)	

Ordinal logistic model based on COVID-19-specific mortality

The overall model regressing on having one or more pulmonary conditions was marginally significant (p-value = 0.083) with an odds ratio (OR) of 1.8205 and 95% confidence interval (CI) of (0.9140, 3.5607). The individual models regressing on each lung comorbidities were also evaluated. Having COPD (OR=1.3022, 95% CI=0.4732, 3.2548, p-value=0.587) and having asthma (OR=0.6613, CI=0.2113, 1.7281, p-value=0.431) did not have a significant effect on the severity of COVID-19 outcomes or death from COVID-19. Having other lung diseases including interstitial lung disease and PH was significantly associated with an increased risk of more severe COVID-19 outcomes and death from COVID-19 (OR=6.6153, CI=2.5944, 17.0795, p-value < 0.001) (Table 1). The multivariate model showed similar results to the single variate (univariate) models, with interstitial lung diseases and PH showing significant association to increased risk of more severe COVID-19 outcomes (OR=6.7819, CI=2.6171, 17.7869, p-value < 0.001) (Table 3).

**Table 3:** Summary of models, death being COVID-19 specific CI: confidence interval; vs.: versus; COPD: chronic obstructive lung disease; ILD: interstitial lung disease; PH: pulmonary hypertension

Models	Odds Ratio	95% CI	P-value
Pulmonary Conditions			
Age	0.9986	0.9783, 1.0198	0.897
Sex: Male vs Female	1.3902	0.7172, 2.7942	0.034
Pulmonary Conditions: Yes vs. No	1.8205	0.9140, 3.5607	0.083
COPD			
Age	0.9999	0.9794, 1.0211	>0.9
Sex: Male vs. Female	1.3129	0.6819, 2.6153	0.425
COPD: Yes vs. No	1.3022	0.4732, 3.2548	0.587
Asthma			
Age	1.0003	0.9802, 1.0213	>0.9
Sex: Male vs. Female	1.2736	0.6583, 2.5476	0.481
Asthma: Yes vs. No	0.6613	0.2113, 1.7281	0.431
Other lung diseases, including ILD and PH			
Age	0.9971	0.9765, 1.0185	0.786
Sex: Male vs. Female	1.1982	0.6097, 2.4301	0.606
Other lung diseases, including ILD and PH: Yes vs. No	6.6153	2.5944, 17.0795	<0.001
Multivariate			
Age	0.9954	0.9743, 1.0173	0.675
Sex: Male vs. Female	1.1502	0.5819, 2.3445	0.692
COPD: Yes vs. No	1.0983	0.3581, 3.0298	0.862
Asthma: Yes vs. No	0.5694	0.1718, 1.5763	0.311
Other lung diseases, including ILD and PH: Yes vs. No	6.7819	2.6171, 17.7869	<0.001

Ordinal logistic model based on all-cause mortality

After switching COVID-19 specific mortality to all-cause mortality, the overall model regressing on having one or more pulmonary conditions was no longer marginally significant (OR=1.5276, CI=0.7734, 2.9525, p-value = 0.213). The models regressing on each lung comorbidities, including COPD (OR=1.1112, 95% CI=0.4057, 2.7556, p-value=0.827) and asthma (OR=0.6044, CI=0.1938, 1.5712, p-value=0.336) did not significantly affect severe COVID-19 outcomes and all-cause mortality. However, having other lung diseases including interstitial lung disease and PH was significantly associated with increased risk of more severe COVID-19 outcomes and all-cause mortality (OR=5.0885, CI=2.0590, 12.5542, p-value <0.001). The multivariate model regressing on having COPD, asthma, and other lung diseases showed similar results to the single variate models, with only interstitial lung disease and PH showing significant association with increased risk of more severe COVID-19 outcomes and all-cause death (OR=5.3101, CI=2.1105, 13.3545, p-value <0.001) (Table 4).

**Table 4:** Summary of models, death being all-cause CI: confidence interval; vs.: versus; COPD: chronic obstructive lung disease; ILD: interstitial lung disease; PH: pulmonary hypertension

Models	Odds Ratio	95% CI	P-value
Pulmonary Conditions			
Age	1.0046	0.9844, 1.0258	0.662
Sex: Male vs. Female	1.2935	0.6760, 2.5542	0.446
Pulmonary Conditions: Yes vs. No	1.5276	0.7734, 2.9525	0.213
COPD			
Age	1.0058	0.9855, 1.0272	0.581
Sex: Male vs. Female	1.2471	0.6546, 2.4484	0.510
COPD: Yes vs. No	1.1112	0.4057, 2.7556	0.827
Asthma			
Age	1.0056	0.9855, 1.0266	0.591
Sex: Male vs. Female	1.1971	0.6248, 2.3613	0.594
Asthma: Yes vs. No	0.6044	0.1938, 1.5712	0.336
Other lung diseases, including ILD and PH			
Age	1.0033	0.9829, 1.0246	0.759
Sex: Male vs. Female	1.1410	0.5893, 2.2708	0.700
Other lung diseases, including ILD and PH: Yes vs. No	5.0885	2.0590, 12.5542	<0.001
Multivariate			
Age	1.0020	0.9811, 1.0239	0.851
Sex: Male vs. Female	1.0972	0.5633, 2.1947	0.788
COPD: Yes vs. No	0.9534	0.3183, 2.5761	>0.9
Asthma: Yes vs. No	0.5533	0.1705, 1.5010	0.278
Other lung diseases, including ILD and PH: Yes vs. No	5.3101	2.1105, 13.3545	<0.001

## Discussion

In patients with HIV, lung diseases have always been a cause of increased morbidity and mortality. A recent global review found that 544 million individuals suffer from chronic lung disease [8], a leading cause of morbidity and mortality [9]. Most chronic lung diseases possess no cure, placing a lifetime of symptoms and health burdens on patients, their families, and caregivers. Many chronic lung diseases are analyzed in disability-adjusted life-years lost due to the condition, and lung diseases rank highly worldwide in years lost of full health [10].

Using different models in our study showed that the severity of COVID-19 outcomes or death was not affected by lung comorbidities, including COPD (which ranks ninth around the globe in years lost) and asthma (which ranks 28th around the world in years lost) [11]. However, other lung diseases, such as interstitial lung disease and PH, were associated with an increased risk of more severe COVID-19 outcomes and death from COVID-19.

Previous studies have shown mixed results. Several studies state that there is little association between the comorbidity status of PLWH and the potential for an increased risk of adverse outcomes and disease severity [12-14]. The use of antiretroviral therapy and a protective effect from the impaired immune reactivity of PLWH have been postulated to contribute to the lack of increased mortality and adverse outcomes in the aforementioned studies; however, a definitive link has yet to be shown [15-17]. Other studies have highlighted the potential association between an increased rate of COVID-19 infections, hospitalizations, and adverse outcomes in PLWH with comorbidities such as advanced age, respiratory diseases (COPD, chronic lung disease), and hypertension [18-25]. A study by Navaneethen et al. found a strong association between PH and all-cause mortality with a log-rank p<0.001 [26].

Findings from this current study highlight the importance of considering specific lung diseases as potential risk factors for poor COVID-19 prognosis in PLWH. The study effectively addresses a notable gap in the existing literature regarding respiratory disease and provides additional evidence regarding the impact of ILD and PH on COVID-19 hospitalization outcomes. Consequently, it emphasizes the need for targeted interventions and comprehensive management strategies explicitly tailored for PLWH with these lung conditions. However, the contrasting results highlight the need for further tailored research on respiratory comorbidities, particularly concerning COVID-19 outcomes in PLWH. By addressing these areas in research, healthcare providers can enhance their ability to deliver optimal care and improve health outcomes in PLWH and associated lung conditions.

We noted sex disparity in our data. Most patients were males (68%, n=146), but after analysis, sex was not significant across the four groups. This suggested that the baseline patient characteristics were balanced. Although most patients had obstructive lung disease (asthma and COPD), obstructive lung disease was not significant across the four groups. This study had a reasonable sample size; however, our study consisting of data from a single healthcare entity in Minnesota, Fairview Minnesota network, can limit the generalization of the results.

## Conclusions

HIV infection can lead to non-infectious pulmonary complications like emphysema, bronchiectasis, interstitial lung diseases, and PH. COVID-19 infection in PLWH and those with underlying pulmonary conditions can lead to increased mortality. Our retrospective study that assessed the impact of superimposed COVID-19 infection on pre-existing lung pathologies in patients living with HIV infection showed increased all-cause mortality and COVID-19-specific mortality in PLWH infection with pre-existing interstitial lung disease or PH. To mitigate this poor outcome, it is imperative that healthcare providers, especially hospitalists/general practitioners and those specializing in infectious disease and pulmonary critical care, find ways to encourage the COVID-19 vaccine uptake among PLWH with underlying interstitial lung disease or PH. Sequel to the paucity of studies with similar results, there is a need for further analysis of how comorbidities, including respiratory comorbidities, may play a role in COVID-19 outcomes for PLWH.
